# Chronic diseases among the elderly in a rural Vietnam: prevalence, associated socio-demographic factors and healthcare expenditures

**DOI:** 10.1186/s12939-015-0266-8

**Published:** 2015-11-17

**Authors:** Jonathan Mwangi, Asli Kulane, Le Van Hoi

**Affiliations:** Global Health, Department of Public Health Sciences, Karolinska Institute, Widerströmska Huset, Tomtebodavägen 18A, Stockholm, Sweden; National Lung Hospital, EVIPNet Vietnam, 463, Hoang Hoa Tham, Ba Dinh, Hanoi Vietnam

**Keywords:** Non communicable diseases, Elderly, Socio-demographic, Health expenditure, Vietnam

## Abstract

**Background:**

Globally, the population of elderly persons is increasing as well as the prevalence of chronic diseases. This change is causing increased healthcare costs to health care systems threatening to push many households into poverty. Low and middle income countries are projected to experience the greatest impact from this change. This study aims to describe the prevalence of common chronic diseases (CCDs) among the elderly in Vietnam, the associated socio-demographic factors and healthcare expenditures.

**Methods:**

This is a cross-sectional study in the FilaBavi demographic surveillance site in Vietnam. 2873 persons over 60 years were randomly sampled. Prevalence of CCDs was reported from study subjects who previously were informed by physicians. Healthcare expenditures were determined from recall of expenses during the last hospital visit. Binomial logistic regression was done to determine the socio demographic predictors of having a CCD or multiple CCDs. Mean healthcare expenditures for the elderly with CCDs and those without CCDs were summarised and compared.

**Results:**

Forty two percent of the elderly were found to have at least one CCD. Joint problems were the most common CCD at 35 %, followed by hypertension at 15 % and chronic bronchitis at 11 %. Being female (OR = 1.51, 95 % CI = 1.03–2.21, *p*-value = 0.036), higher education (OR = 2.54, 95 % CI = 1.13–5.74, p-value = 0.025) and having advanced age (OR = 1.92, 95 % CI = 1.22–3.00, *p*-value = 0.005), were associated with common chronic diseases in the elderly. Outpatient healthcare expenditures were found to be significantly higher for the elderly with CCDs than those without CCDs.

**Conclusions:**

Higher education and being female are important key predictors of having a CCD, while wealth quintile is a predictor of multimorbidity, in the elderly. Healthcare expenditures for outpatient health services are higher for elderly persons with CCDs and these costs should be targeted when planning for financial protection.

## Background

Chronic diseases are described by the World Health Organisation, as diseases of long duration and slow progress. They include non communicable diseases such as heart diseases, stroke, cancer, chronic respiratory diseases and diabetes [1]. The definition of an elderly person remains a topic for debate as it varies in different societies. It may be associated with the age when a person can start to receive pension benefits. In the high income countries the cut off point is 65 years while the United Nations has an arbitrary cut off of 60 years and above.

The rise in the prevalence and significance of chronic diseases has been attributed to the complex interaction between health, economic growth and development. These diseases have continued to exert tremendous demand on the social welfare and health systems. Chronic diseases have substantially contributed to rising healthcare costs [2]. They are also responsible for contributing to decreased productivity in the workplace, prolonged disability and diminished resources within families thereby threatening to push millions into poverty [3]. This is in addition to the physical anguish and reduced quality of life they cause to the patients and their caregivers. Globally chronic diseases currently account for up to 2 in every 3 deaths reported [4], with 80 % of the deaths coming from low and middle income countries [3]. It is also projected that by 2020 the South East Asia and Western Pacific region will have the greatest number of deaths from chronic diseases globally [5].

The global population is ageing, with total number of elderly persons increasing globally. This has been attributed to improvement in healthcare and nutrition. Between 1970 and 2010 the life expectancy at birth rose from 56.4 years to 67.5 years for males and 61.2 years to 73.3 years for females [6]. Globally the proportion of population aged 60 years and above was estimated at 11 % in 2007 and set to rise to over 20 % by 2050, a process that is described as irreversible [7]. On the other side, increased healthy life expectancy has been accompanied by an increase in the number of healthy years lost to disability due to chronic diseases as ageing increases vulnerability to chronic diseases [8]. This growing elderly population with chronic diseases presents a challenge to healthcare systems due to increased healthcare expenditure [6, 8]. In Taiwan, expenditures due to chronic diseases among the elderly were up to 90 % of the health expenditure for the elderly, and 5 times higher compared to expenditures for those without chronic diseases [9]. Between these two factors, presence of chronic diseases has been described to have a higher influence on healthcare expenditure than ageing [10]. The increases in healthcare expenditures from the health transition, at household level may expose vulnerable individuals or households to high medical expenditures leading to poverty [11, 12].

In Vietnam, the total population has been growing, currently at 87 million, with 6 % being over 65 years old. Crude mortality rates have been falling with a reducing fertility rate currently at 1.9. This has resulted in an ageing population, with the age group over 60 years growing fastest and projected to be 26 % of the population by 2050. The proportion of chronic diseases in total mortality increased from 41.1 % in 1986 to 61.6 % in 2006. In addition, the incidence of chronic diseases has been shown to increase rapidly especially among elderly people [13]. The most common chronic diseases among the elderly in Vietnam include cardiovascular diseases, joint problems, and chronic pulmonary diseases [14]. Presence of a chronic illness among the rural elderly, in Vietnam has also been shown to be a determinant for the need of care [15] and higher probability of catastrophic health expenditures (when a household’s out of pocket health payments equal or exceed 40 % of household income after basic need s have been met) [16]. Other factors include, coming from a poor household and having no health insurance [16]. The elderly in rural Vietnam are thus a vulnerable population, and efforts to offer them financial protection through health care funds for the poor policy have had limited success [17]. Our study aims to describe the prevalence of common chronic diseases (CCDs) among the elderly to monitor the trend of CCDs and describe the healthcare expenditures associated to having CCDs.

## Methods

This is a cross sectional survey study investigating prevalence of CCDs and associated socio-demographic factors and healthcare expenditures. It was a component of a larger study conducted in 2007 in the Fila Bavi Demographic Surveillance site in rural Vietnam. Previously analysed and published papers from the survey data include, remaining life expectancy among older people [18], health-related quality of life and its determinants among older people in Vietnam [19], willingness to pay for formal options of care for community dwelling older people in rural Vietnam [20], and Elderly care activities of daily living in rural Vietnam and its determinants [15].

In 2007, 2240 households with 2873 people aged 60 years and older, were randomly selected from the FilaBavi Demographic Surveillance System (DSS). This represented 50 % of the households with elderly people followed by Fila Bavi. Data was collected by way of questionnaires designed for the study administered to households with elderly persons, through face to face interviews with the elderly or household representatives.

Respondents were asked if they had any of the following common chronic diseases, previously diagnosed by a physician; hypertension, diabetes, cancer, arthritis/osteoarthritis, stroke, angina-pectoris, chronic bronchitis, and cataract. The total number of chronic disease was determined by summation of positive responses for each disease. For each disease, the percentage of positive responses of all responses for that disease was used to determine the prevalence. The prevalence was further aggregated by taking number of positive responses for each elderly person to determine how many suffered from multiple diseases (multimorbidity). These responses were aggregated as those with no disease (no positive response), those with one disease, two diseases and those with 3 or more diseases.

Socio-demographic factors of interest in the study were sex, age, marital status, level of education, whether the elderly person was the head of the household, ethnicity, religion, occupation status and economic situation of the household from which a respondent came. Level of education was categorized as illiterate, read and write, primary/secondary school level, high school level and above high school level. These were self reported education levels. The data was further aggregated to those who could read and write only and those who had primary or secondary education or above. Occupation status was categorized as having a job/no job, farmer, worker, officer, or in business. Long term economic situation for the household was determined by classifying the household into 5 wealth quintiles as poorest, poor, middle, richer, richest. Hierarchy among households in FilaBavi based on basic assets was used to determine household wealth quintiles [15]. The 2006–2010 national poverty line for rural areas, based on a monthly per capita income equivalent to VND 200,000 (12.5 American dollars) was used to determine the household poverty status [15].

Healthcare expenditures were determined by asking the respondent to recall the amount of money spent during their last illness episode with a visit to a health facility. Costs were categorised as either incurred during an inpatient visit or for an outpatient visit. Expenditure items include costs for diagnosis and treatment, travel, food and drink, communication and income lost of the accompanying relatives. These costs were reported as direct costs or indirect costs. Direct costs constituted payments made for diagnosis, treatment and medications. Indirect expenditures were those expenses incurred for transport, food and drink, communication as well as income lost during the visit. Mean costs were compared for two groups, those having CCDs and those not having CCDs. To complement healthcare costs, health service utilization was also described, for both inpatient and outpatient.

### Statistical analysis

SPSS version 21 and STATA version 12 software were used for the descriptive and analytical statistical analysis. Socio demographic characteristics of the study population were summarised into proportions. Prevalence of CCDs with corresponding 95 % confidence intervals was computed. Binary logistic regression using the backward stepwise elimination likelihood ratio method was performed. Having a CCD or no CCD, as well as having one CCD or having more than one CCD as the outcome variables, and all socio-demographic factors, were the predictor variables. The significance level set at *p* < 0.05.

Health care costs were categorised as direct and indirect costs, for both inpatient and outpatient services. They were then summarised using simple means, for those with CCDs and those without CCDs. The mean costs were compared using independent sample *t*-test. The significance level was set at *p* < 0.05.

Health service utilization was also described for two groups, those with CCDs and those without CCDs and utilization rates compared using independent samples *t*-test, *p* < 0.05.

Ethical approval for the FilaBavi demographic surveillance system, including data on socioeconomic status, was given by the Research Ethics Committee at Umeå University, Sweden (reference number 02–420). The present study was also approved by the Research Ethics Committee at Hanoi Medical University (reference number 51/HMU-RB).

## Results

The socio-demographic characteristics of the study sample are given in Table 1. Proportion of males was lower than that of females. Majority of them was young-old people. Almost one-third of them were widowed or separated. Just over three fifths had primary/secondary education and above. Percentage of elderly who were household heads is higher than those who were household members. Most of older people was belonged to the Kinh – a major ethnic group in Vietnam. The households were equally distributed across the 1st, 2nd, 3rd and 4th wealth quintile, at approximately 21 % for each wealth quintile. When classified by the national poverty line, 85 % of the households were above the national poverty line. Majority of elderly was still working at old ages.

**Table 1:** Socio-demographic characteristics of the study sample

				95 % C.I	
		*n*	Percent	Lower	Upper
Sex	Male	476	41.6	38.7	44.6
	Female	669	58.4	55.4	61.3
Age	60–65	413	36.1	33.4	38.9
	65–69	313	27.3	24.7	29.8
	70–74	224	19.6	17.2	21.9
	75–79	147	12.8	11	14.9
	80–84	34	3	2	4
	85+	14	1.2	0.6	1.8
Marital status	Married	786	68.6	65.9	71.4
	Separated	16	1.4	0.8	2.1
	Widowed	326	28.5	25.8	31.1
	Single	17	1.5	0.9	2.3
Education	Illiterate	99	8.6	7.1	10.4
	Read and write only	348	30.4	27.8	33.2
	Primary/secondary	607	53	50	55.9
	Highschool	42	3.7	2.6	4.9
	Above highschool	49	4.3	3.1	5.5
Household head	No	450	39.3	36.6	42.2
	Yes	695	60.7	57.8	63.4
Ethnicity	Kinh	1069	93.4	91.8	94.8
	Muong	76	6.6	5.2	8.2
Religion	None	1096	95.7	94.5	96.9
	Buddism	6	0.5	0.2	1
	Christian	43	3.8	2.6	4.9
Wealth QT	1	237	20.7	18.3	23
	2	235	20.5	18.2	22.8
	3	241	21	18.6	23.3
	4	239	20.9	18.4	23.3
	5	193	16.9	14.7	19
NPL	Above NPL	976	85.2	83.1	87.2
	Below NPL	169	14.8	12.8	16.9
Working status	No working	115	10	8.3	11.9
	Still working	1030	90	88.1	91.7

Prevalence of self reported CCDs is as presented in Table 2. Joint problems were the commonest reported CCD, followed by hypertension, chronic bronchitis and cataracts. Cancer and diabetes had very few cases reported, less than 1 % for each. Noted also was the gender difference in the prevalence of the self reported CCDs. Women had twice the prevalence of osteoarthritis compared to men. For self reported chronic bronchitis, men had a higher prevalence compared to women. We also aggregated the prevalence by number of CCDs per person (Table 3). Just over two-fifths of the respondents reported having at least one CCD. Percentage of having more concurrent CCDs sharply decreased when the number of diseases increased.

**Table 2:** Prevalence of self reported CCDS

	CCD prevalence		
	Male	Female	Total
	Percent (95 % c.i)	Percent (95 % c.i)	Percent (95 % c.i)
Hypertension	16 (13–18)	14 (13–16)	15 (13–16)
Diabetes	0 (0–1)	0 (0–1)	0 (0–1)
Arthritis/osteoarthritis	23 (21–26)	41 (39–43)	35 (33–36)
Stroke	3 (2–5)	1 (1–2)	2 (2–3)
Angina pectoris	5 (3–6)	3 (2–4)	4 (3–4)
Chronic bronchitis	15 (12–17)	9 (7–10)	11 (10–12)
Cancer	0 (0–0)	0 (0–0)	0 (0–0)
Cataract	3 (2–5)	6 (5–7)	5 (4–6)

**Table 3:** Aggregated self reported CCD prevalence

	95 % C.I			
	n	Percent	Lower	Upper
None	1662	57.9	56.1	59.7
One	841	29.3	27.8	31.1
Two	265	9.2	8.2	10.3
3 or more	100	3.5	2.8	4.1

**Table 4:** Mean or Proportion of cases in each category

		No CCD	With CCD
Age		71.88	72.89
Sex	Male	37.30 %	35.80 %
	Female	62.70 %	64.20 %
Marital status	Married	55.90 %	53.10 %
	Separated	1.00 %	1.40 %
	Widowed	41.90 %	44.10 %
	Single	1.30 %	1.30 %
Education	Illiterate	18.20 %	17.90 %
	Read and write only	34.60 %	36.10 %
	Primary/secondary	40.60 %	37.00 %
	High school	2.70 %	3.30 %
	Above high school	3.90 %	5.70 %
Head of household	No	48.00 %	47.80 %
	Yes	52.00 %	52.20 %
Ethnicity	Kinh	96.50 %	95.90 %
	Muong	3.50 %	3.90 %
	Other	0.00 %	0.20 %
Religion	None	96.00 %	96.00 %
	Buddism	1.60 %	0.70 %
	Christian	2.40 %	3.20 %
Wealth Qt.	1	17.70 %	16.80 %
	2	17.70 %	16.60 %
	3	23.60 %	20.10 %
	4	21.60 %	23.10 %
	5	19.30 %	23.40 %
NPL	Above NPL	84.60 %	85.80 %
	Below NPL	15.40 %	14.20 %
Working status	No job	10.80 %	9.40 %
	Have job	89.20 %	90.60 %

Mean and proportions for each category are provided in Tables 4 and 5. Results of the binomial logistic regression are presented in Tables 6 and 7. Sex, education and age-group were found to be significant predictors of having CCDs. Females were 1.5 more time likely to have a CCD compared to males. Those having high school education had 2.5 more time likelihood of having a CCD compared to those who were illiterate. Those aged between 75–79 years were 1.9 more times likely to have a CCD compared to those aged 65 years and below.

**Table 5:** Mean or Proportion of cases within each category

		No CCD	1 CCD	≥2 CCDs
Age		71.88	72.83	73.04
Sex	Male	37.30 %	35.40 %	36.70 %
	Female	62.70 %	64.60 %	63.30 %
Marital status	Married	55.90 %	52.70 %	54.00 %
	Separated	1.00 %	1.30 %	1.70 %
	Widowed	41.90 %	44.40 %	43.50 %
	Single	1.30 %	1.60 %	0.80 %
Education	Illiterate	18.20 %	18.90 %	15.60 %
	Read and write only	34.60 %	34.80 %	38.90 %
	Primary/secondary	40.60 %	38.00 %	34.50 %
	High school	2.70 %	2.70 %	4.70 %
	Above high school	3.90 %	5.50 %	6.30 %
Head of household	No	48.00 %	48.20 %	46.80 %
	Yes	52.00 %	51.80 %	53.20 %
Ethnicity	Kinh	96.50 %	95.10 %	97.80 %
	Muong	3.50 %	4.60 %	2.20 %
	Other	0.00 %	0.20 %	0.00 %
Religion	None	96.00 %	95.80 %	96.40 %
	Buddism	1.60 %	1.00 %	0.30 %
	Christian	2.40 %	3.20 %	3.30 %
Wealth Qt.	1	17.70 %	16.20 %	18.40 %
	2	17.70 %	16.60 %	16.40 %
	3	23.60 %	21.90 %	16.20 %
	4	21.60 %	22.90 %	23.30 %
	5	19.30 %	22.40 %	25.80 %
NPL	Above NPL	84.60 %	86.10 %	85.20 %
	Below NPL	15.40 %	13.90 %	14.80 %
Working status	No job	10.80 %	8.00 %	12.90 %
	Have job	89.20 %	92.00 %	87.10 %

**Table 6:** Logistic regression analysis for having an CCD in relation to socio-demographic factors

			95 % CI		
Socio-demographic characteristic		OR	lower	upper	*p*-value
Sex	*Ref*. Male	1.00			
	Female	1.51	1.03	2.21	0.036
Education	*Ref*. Illiterate	1.00			
	Read and write	1.44	0.89	2.34	0.141
	Primary/secondary	1.38	0.83	2.27	0.213
	Highschool	2.54	1.13	5.74	0.025
	Above highschool	1.93	0.89	4.18	0.096
Age	*Ref* < 65	1.00			
	65–69	1.24	0.91	1.70	0.171
	70–74	1.32	0.92	1.92	0.129
	75–79	1.92	1.22	3.00	0.005
	80–84	0.66	0.28	1.58	0.35
	85+	0.51	0.10	2.63	0.417

**Table 7:** Logistic regression^a^ analysis for having one CCD or more than one CCDs in relation to socio-demographic factors

		OR	95 % C.I.		
			Lower	Upper	*p*-value
Step 1a	NPL(1)	1.005	0.496	2.036	0.99
Step 2a	age_group(1)	0.742	0.404	1.362	0.335
	age_group(2)	0.859	0.434	1.698	0.661
	age_group(3)	0.835	0.397	1.758	0.635
	age_group(4)	0.753	0.167	3.386	0.711
	age_group(5)	1.88	0.229	15.419	0.556
Step 3a	Religion(1)	0	0		1
	Religion(2)	0.65	0.175	2.41	0.52
Step 4a	Education(1)	1.1	0.452	2.681	0.833
	Education(2)	0.904	0.364	2.246	0.828
	Education(3)	2.103	0.555	7.959	0.274
	Education(4)	0.607	0.142	2.589	0.5
Step 5a	Head of household(1)	0.822	0.46	1.468	0.508
Step 6a	Sex(1)	0.747	0.439	1.272	0.283
Step 7a	Marital status(1)	4.808	0.841	27.502	0.078
	Marital status(2)	1.202	0.735	1.968	0.463
	Marital status(3)	1.106	0.205	5.97	0.906
Step 8a	Ethnicity(1)	0.593	0.231	1.526	0.279
Step 8a	Have no job(1)	0.565	0.275	1.158	0.119
Step 10b	Wealth_quintile(1)	0.669	0.341	1.311	0.241
	Wealth_quintile(2)	0.362	0.173	0.758	0.007
	Wealth_quintile(3)	0.776	0.414	1.453	0.428
	Wealth_quintile(4)	0.722	0.374	1.396	0.334

^a^The selection process of the backward stepwise in the regression is shown in the table below, from step 1 to 9, nine non-significant variables were eliminated and their respect OR, CI and p-value at the time of removal are listed. The final step retains only one explanatory variable in the model, i.e., *wealth quintile*

Among the patients with CCDs, multimorbidity was found to be associated with wealth quintile. The risk of having more than one CCD for patients in the third wealth quintile (middle), was only 36 % in comparison to the reference group of being in the first wealth quintile (poorest).

Results for health service utilization (Table 8) showed a higher utilization rate for the elderly with CCDs. Results for healthcare costs from those who responded were summarised as simple means (Table 9). All mean expenditures for inpatients were higher, at least 4.5 times, than that of the same items for outpatients. Mean expenditures for outpatient for those with CCDs was higher than that for those without CCDs.

**Table 8:** Comparison of health service utilization between those without CCDs and those with CCDs

Independent Samples Test						
		N	total	%	% difference	*p*- value
Elderly that sought outpatient sevices during the last 12 months	No CCD	1041	1662	63 %	−19 %	<0.001
	With CCD	965	1206	82 %		
Elderly that sought inpatient serives during the last 12 months	No CCD	81	1662	5 %	−8 %	<0.001
	With CCD	157	1206	13 %

**Table 9:** Summary of healthcare expenditure^a^ in VND (currency)

				95 % C.I	
Outpatient costs		n	Mean	Lower	Upper
No CCD	Diagnosis and treatment cost	798	94,523.81	77,040.15	114,689.00
With CCD	Diagnosis and treatment cost	782	142,686.70	114,126.10	173,664.40
No CCD	total_indirect_outpatient	818	78,722.50	15,024.70	203,987.80
With CCD	total_indirect_outpatient	789	32,528.52	24,066.90	42,111.31
No CCD	total cost for outpatient	846	107,005.90	88,316.00	129,847.00
With CCD	total cost for outpatient	805	194,027.30	154,502.60	239,183.90
Inpatient costs					
No CCD	Diagnosis and treatment cost	36	559,722.20	198,448.70	1,030,526.00
With CCD	Diagnosis and treatment cost	69	638,043.50	310,406.10	1,026,176.00
No CCD	total_indirect_inpatient	39	367,512.80	238,489.10	544,145.00
With CCD	total_indirect_inpatient	76	346,723.70	223,406.10	517,762.40
No CCD	total cost for inpatient	37	837,513.50	446,858.50	1,373,579.00
With CCD	total cost for inpatient	75	1,081,707.00	638,593.80	1,634,392.00

^a^Average expenditures were estimated from only those had responded on their healthcare expenditure

## Discussion

Overall, there was an increase of the prevalence of chronic diseases in the elderly with 42.1 % reporting at least one chronic disease compared to 39.6 previously reported [14]. This could be a pointer to improving healthcare system that is allowing access to chronic care, allowing people to live longer with chronic diseases. However it is also noted that in this study, the prevalence of CCDs was lower from 47.7 % for the 75–79 age group to 42 % for those aged over 85 years. This may indicate mortality from CCDs, leaving those without CCDs to live longer; consistent with a previous conclusion that mortality from CCDs reduces the life expectancy [21]. This interaction, of increased life expectancy and increased mortality from chronic diseases is expected to continue as the country goes through the transition. But perhaps a key issue to note is that mortality from CCDs is higher among those with low socioeconomic status, and low education level [18, 21–23], while life expectancy rises among those with higher wealth status, leading to inequalities in life expectancies [18]. It is this growing inequality that should remain the focus area of policies that aim to bridge this gap.

Sex, education and age have been described in studies before as predictors of chronic illness in Vietnam [24–26] and other Asian countries [27]. In this study, men generally had higher prevalence of cardiovascular diseases. They also had a higher prevalence of chronic bronchitis. This higher prevalence among the men is probably attributable to the prevalence of higher risk factors for these diseases among the men [14]. Females had a much higher prevalence of joint problems at 41 % compared to the males at 23 % and this increase in the overall odds of females having a CCD compared to males needs further studies to be done.

Level of education, has been found to have an inverse relationship with the prevalence of chronic illness [14, 28]. In contrast, education has a positive relation to good health outcomes as well as reduced mortality rates from non communicable diseases [18, 21, 22]. This would in turn be evident from higher prevalence of chronic illness in those with higher education status, in keeping with our study. This further highlights education as a factor of inequity in healthcare [21, 23].

Healthcare expenditures were higher for the patients with CCDs compared to those without. This is in contrast to findings from a study that looked at household out of pocket payments in FilaBavi. The study found expenditures on communicable illness to predominate in household health expenditures [29]. However our study was focused on healthcare expenditures for the elderly as opposed to the entire household and found expenditures due to CCDs to be higher. In India expenditures due to CCDs were up to twice the costs incurred for other diseases [30]. In Vietnam chronic diseases have been described as a determining factor in household health expenditure [14] and constant fees for health services may eventually push households into poverty [29]. In this study, this could be a possibility from the increased outpatient fees paid by the patients with chronic diseases, as well as seeking outpatient services at a higher frequency compared to those no CCD [16].

### Limitations

Recall bias and recall errors are possible limitation to this study. In the study we relied on self reported CCDs that had been physician confirmed to minimise the errors. In comparing the mean costs for the two groups, those having CCDs and those not having CCDs the methodology employed may not give a direct link to the difference in costs being associated with CCDs. Also not all respondents could recall their healthcare expenditures.

From the cross sectional study design, we measure the prevalence of disease or outcome at a point in time and it may be difficult to assess association of the discussed risk factors unless the sequence of events is certain. That is, the exposure precedes the outcome. However, our findings provide results from which we can generate hypothesis for future research.

## Conclusion

Ageing of the population will continue, as an outcome of falling birth rates, improved healthcare systems and socioeconomic status of the population. Prevalence of chronic diseases is expected to increase in this growing elderly population. Education remains a key determinant of good health outcomes in populations and more investment in education to the population should continue. In the broader context of social determinants of health, benefits of sustained economic growth should offer education and employment opportunities to households, as way of enhancing equity in the society in addition to improved healthcare services.

In the face increasing prevalence of chronic diseases and improving healthcare, increased access to healthcare will come with increased household expenditures for healthcare. In particular, our study identifies the elderly persons with chronic diseases as having a higher financial burden compared to those without CCDs thus advocating further attention to this group when offering financial protection. Catastrophic healthcare costs have been described, not commonly as single rare events but rather as continuous costs that eventually grind households into poverty. From our study, this risk is evident from the higher mean costs for outpatient care services for patients with CCDs compared to those without CCDs.
